# Maternal and Perinatal Outcomes in Hypertensive Disorders of Pregnancy and Factors Influencing It: A Prospective Hospital-Based Study in Northeast India

**DOI:** 10.7759/cureus.13982

**Published:** 2021-03-18

**Authors:** Subrat Panda, Rituparna Das, Nalini Sharma, Ananya Das, Prakash Deb, Kaushiki Singh

**Affiliations:** 1 Obstetrics and Gynaecology, North Eastern Indira Gandhi Regional Institute of Health & Medical Sciences (NEIGRIHMS), Shillong, IND; 2 Obstetrics and Gynecology, North Eastern Indira Gandhi Regional Institute of Health & Medical Sciences (NEIGRIHMS), Shillong, IND; 3 Anesthesiology and Critical Care, North Eastern Indira Gandhi Regional Institute of Health & Medical Sciences (NEIGRIHMS), Shillong, IND

**Keywords:** hypertensive disorders of pregnancy, preeclampsia, eclampsia, gestational hypertension, maternal mortality, perinatal outcome, proteinuria, antenatal screening

## Abstract

Introduction

Hypertensive disorders frequently complicate pregnancy and contribute substantially to maternal and perinatal morbidity and mortality. Identification of risk factors for hypertensive disorders of pregnancy (HDP) can help determine the particular patient group which requires appropriate intervention.

Methods

This prospective cross-sectional hospital-based study conducted from January 2016 to January 2019 included all pregnant women beyond 20 weeks of gestation complicated by HDP. The objectives were to determine the incidence of HDP and associated maternal and perinatal mortality and morbidity rates along with factors influencing it. Data collected were entered in Microsoft Excel (Microsoft Corporation, Redmond, WA) and analyzed with the Statistical Package for the Social Sciences (SPSS) software version 21 (IBM Corp. Armonk, NY).

Results

In our study, out of 5460 deliveries, 402 (7.4%) cases had HDP, 27.6% had gestational hypertension, 27.6% had mild preeclampsia, 33.6% had severe preeclampsia, and 11.2% had eclampsia. Fifty-four (13.4%) cases required admission in the intensive care unit and 12 (2.9%) ended in maternal deaths. The cause of maternal mortality was cerebral hemorrhage in eight (66.6%) cases and pulmonary edema in four (33.3%) cases. All maternal deaths occurred in women with severe preeclampsia and eclampsia and eclampsia was significantly higher. Maternal deaths were more when systolic blood pressure (SBP) was ≥ 160mmHg, diastolic blood pressure (DBP) was ≥ 110mmHg, significantly more with 3+ proteinuria, but no association was found with age, parity, booking status, socio-economic status, gestational age, or mode of delivery. All mothers with HDP received treatment with antihypertensives. There were 60 (14.9%) cases of perinatal mortality. Perinatal deaths were more in unbooked cases and preterm HDP, significantly more with SBP ≥160 mmHg, DBP ≥110 mmHg and ≥2+proteinuria, but no association was found with parity or mode of delivery. Besides mortality, there was a significant burden of maternal and perinatal morbidity, which was more in women with severe preeclampsia and eclampsia.

Conclusion

Routine antenatal screening for HDP in all pregnant women with appropriate and timely interventions in women at risk may help reduce HDP-related maternal and perinatal morbidity and mortality.

## Introduction

Hypertensive disorders complicate 5% to 10% of all pregnancies and form a lethal trio with hemorrhage and infection [1]. A variation in maternal and perinatal morbidity and mortality has been observed worldwide, which could be the result of differences in the socio-economic background of nations as well as the quality of obstetric care delivered to these women. A confidential inquiry into maternal deaths in the UK found hypertensive disorders to be the second leading direct cause of maternal mortality [2]. In the USA, 7% to 15% of all pregnancies were affected by hypertensive disorders and were associated with up to 22% of all perinatal mortality and 30% of all maternal mortality [3]. A study in India in 2006 reported the incidence of hypertensive disorders complicating pregnancy as 5.38% with preeclampsia, eclampsia, and HELLP (hemolysis, elevated liver enzymes, and low platelet count) syndrome accounting for 44%, 40%, and 7% of cases, respectively [4]. Maternal and perinatal deaths have been reported to be 5.5% and 37.5%, respectively [4]. Maternal mortality in women with eclampsia is very high in India, ranging from 8%-14% [5]. With proper antenatal care, early recognition of preeclampsia, and timely intervention, the development of eclampsia and its associated complications can be prevented. However, in few patients where eclampsia develops without any preceding features of preeclampsia, the proper management of eclampsia rather than its prevention is vital in improving maternal and fetal outcomes. Though maternal mortality is decreased in developed countries, it is still high in developing countries and is mainly related to eclampsia, accidental hemorrhage, pulmonary edema, disseminated intravascular coagulation, acute renal failure, and HELLP syndrome [6]. Perinatal mortality, however, continues to be high even in developed countries and is reported to be up to 20% in the developing countries, half of which are stillborn [6]. This study was conducted in our institute to determine the incidence of hypertensive disorders of pregnancy and associated maternal and perinatal mortality and morbidity rates, along with the factors influencing it.

## Materials and methods

This was a prospective, cross-sectional, hospital-based study conducted on women with pregnancies complicated by hypertensive disorders in a teaching hospital in northeast India from January 2016 to January 2019. The criteria for defining different types of hypertension were:

- Preeclampsia was diagnosed in women with a blood pressure of 140/90 mmHg or above on two occasions at least four hours apart after 20 weeks of gestation with proteinuria or in those without proteinuria by the presence of thrombocytopenia (less than 100,000/microlitre), impaired liver function (serum liver transaminases to twice the normal level), new-onset renal insufficiency (serum creatinine more than 1.1 mg/dL or doubling of serum creatinine without any other renal pathology), presence of pulmonary edema, or new-onset visual disturbances or cerebral symptoms. Severe preeclampsia was diagnosed where the blood pressure was 160/110 mmHg and above besides thrombocytopenia, progressive renal dysfunction, impaired liver function, pulmonary edema, and new-onset cerebral or visual disturbances, as already described, with the presence of severe and persistent right upper quadrant or epigastric pain not responsive to medication and other causes excluded. The rest were classified as having mild preeclampsia.

- Eclampsia was diagnosed where a seizure developed in women with hypertension, which was not attributed to any other cause.

- Gestational hypertension was diagnosed in women with hypertension beyond 20 weeks of gestation in the absence of proteinuria or the systemic findings of preeclampsia.

- Chronic hypertension was diagnosed in women with hypertension prior to pregnancy or prior to 20 weeks of gestation.

The presence of proteinuria was detected using a dipstick and classified into 1+/2+/3+. The diagnosis of HELLP syndrome was based on the presence of hemolysis with increased serum lactate dehydrogenase level (> 600 U/L), serum aspartate transaminase (≥70 U/L), and platelet count <100 x 109/L according to the Tennessee Classification System.

All pregnant women beyond 20 weeks of gestation complicated by hypertensive disorders of pregnancy (HDP), both booked and un-booked, and admitted to our institute were included in the study. Pregnancies with chronic hypertension, associated medical complications like anemia, diabetes, vascular or renal disease, multiple pregnancies, fetal growth restriction (FGR) in a previous pregnancy, and a previous history of convulsions secondary to other medical causes were excluded from the study. Women from all socio-economic classes were selected irrespective of their parity and consanguinity. A detailed history of present and previous pregnancies, including last menstrual period, expected date of delivery, and period of gestation were recorded in the proforma.

Prompt treatment was initiated in all women. Women with hypertension received antihypertensive therapy and all women with severe preeclampsia and eclampsia were administered magnesium sulfate according to the Pritchard regimen. All other clinical management, such as decisions regarding induction of labor, mode of delivery, fetal monitoring, etc., was done as per departmental protocol and standard operating procedure.

The primary objective of our study was to determine the incidence of hypertensive disorders in the north-eastern pregnant population. The secondary objectives were to evaluate the maternal and perinatal mortality rates along with the factors influencing them. The association of adverse maternal and perinatal outcomes with the type of hypertensive disorder, degree of hypertension, degree of proteinuria, booking status of the women, gestational age, mode of delivery, and demographic variables like age, parity, and socioeconomic status were studied. Data collected were entered in Microsoft Excel (Microsoft Corporation, Redmond, WA) and analyzed with the Statistical Package for the Social Sciences (SPSS) software version 21 (IBM Corp. Armonk, NY). The chi-square test was used to compare the categorical variables. Based on relative risk calculation, a p-value of less than 0.007 was considered statistically significant.

## Results

In our study, 5460 deliveries were conducted in the study period; of which, 402 (7.4%) cases had HDP. Twenty-seven point six (27.6%) of them had gestational hypertension, 27.6% had mild preeclampsia, 33.6% had severe preeclampsia, 11.2% had eclampsia, and 12 (2.9%) ended in maternal deaths. The cause of maternal mortality was cerebral hemorrhage and pulmonary edema with mortality due to cerebral hemorrhage being significantly higher (p-value 0.0013). All the maternal deaths occurred in women with severe preeclampsia and eclampsia. Maternal death in women with eclampsia was significantly higher than those with severe preeclampsia (p-value 0.0008). Also, maternal deaths were more in women with systolic blood pressure (SBP) ≥160 mmHg and diastolic blood pressure (DBP) ≥110 mmHg than in those with lower blood pressure, but the difference was not statistically significant. Maternal death was also significantly higher in women with 3+ proteinuria (p-value 0.0013). There was, however, no significant association of age, parity, socioeconomic status, mode of delivery, or the booking status of the women with maternal mortality (p-value non-significant). Term or preterm development of hypertensive disorder had no significant association with maternal death (p-value 0.6827). All mothers with HDP received treatment with antihypertensives. In our study, 1.9% cases had HELLP syndrome, 3.7% cases had pulmonary edema, 3.9% cases had a cerebral hemorrhage, 0.7% cases had acute renal failure (ARF), 7.5% cases had abruption placenta, and 5.7% cases had postpartum hemorrhage (PPH). Fifty-four (54; 13.4%) cases required intensive care unit (ICU) admission. All the mothers who had complications requiring ICU care had either severe preeclampsia or eclampsia. ICU admission was significantly more in women with eclampsia than severe preeclampsia (p-value 0.0004) (Table 1). Twelve (12; 22.2%) of those shifted to the ICU ended in maternal mortality.

**Table 1:** Maternal complications HELLP: hemolysis, elevated liver enzymes, and low platelet count; ARF: acute renal failure; PPH: postpartum hemorrhage; ICU: intensive care unit

Type of HDP	No. of cases	% of cases	HELLP N (%)	Pulmonary edema N (%)	Cerebral hemorrhage N (%)	ARF N(%)	Abruptio placentae N (%)	PPH N (%)	ICU admission N (%)
Gestational hypertension	111	27.6						2 (1.8%)	
Mild preeclampsia	111	27.6					7 (6.3%)	6 (5.4%)	
Severe preeclampsia	135	33.6	8 (5.9%)	8 (5.9%)	8 (5.9%)	2 (1.5%)	18 (13.3%)	12 (8.9%)	32 (23.7%)
Eclampsia	45	11.2		7 (15.6%)	8 (17.8%)	1 (2.2%)	5 (11.1%)	3 (6.7%)	22 (48.9%)
Total	402	7.4%	8 (1.9%)	15 (3.7%)	16 (3.9%)	3 (0.7%)	30 (7.5%)	23 (5.7%)	54 (13.4%)

There were 60 (14.9%) cases of perinatal mortality. Perinatal mortality was significantly higher in women with eclampsia and severe preeclampsia (p-value <0.0001) and more so in eclampsia than in severe preeclampsia (p-value <0.0001). Women with SBP ≥160 mmHg and DBP ≥110 mmHg had more perinatal deaths as compared to those with lower blood pressure (p-value 0.0011 and 0.0003, respectively). Perinatal mortality was statistically higher in women with increasing proteinuria (p-value <0.0001) with 2+ or 3+ proteinuria having significantly more perinatal deaths as compared to trace or 1+ proteinuria (p-value 0.0011). Perinatal mortality was significantly higher in young as well as elderly mothers (p-value 0.0001). Also, unbooked cases and women with preterm HDP had more perinatal deaths though not statistically significant (p-value 0.0113 and 0.0327, respectively). Parity, socio-economic status, and mode of delivery had no significant association with perinatal death (p-value 0.2408, 0.441, and 0.2278, respectively). See Table 2. One-hundred fifty-six (156; 38.8%) babies delivered were healthy at birth, 102 (25.3%) had preterm births with respiratory distress syndrome (RDS), 33 (8.2%) had preterm births and FGR, 27 (6.7%) had preterm births and hypoxia, three (0.74%) had term births with FGR, 15 (3.7%) had term births with FGR and hypoxia, six (1.5%) had term births with FGR and meconium aspiration syndrome (MAS), 33 (8.2%) had intrauterine fetal demise (IUFD), and 27 (6.7%) had neonatal deaths. See Table 3.

**Table 2:** Maternal and perinatal mortality and associated factors HDP: hypertensive disorders of pregnancy; SBP: systolic blood pressure; DBP: diastolic blood pressure

Factors		Maternal mortality N(%)	P-value	Perinatal mortality N (%)		P-value
Cause	Pulmonary edema	4 (26.6%)	0.0013			
	Cerebral hemorrhage	8 (50%)				
Type of HDP	Mild preeclampsia (111)	0	0.0008	3 (2.7%)		<0.0001 [<0.0001 (between severe preeclampsia & eclampsia)]
	Severe preeclampsia (135)	4 (2.9%)		33 (24.4%)		
	Eclampsia (45)	8 (17.7%)		24 (53.3%)		
SBP (in mmHg)	≥160 (150)	11 (7.3%)	0.0649	39 (26%)		0.0011
	<160 (252)	1 (0.03%)		21 (8.3%)		
DBP (in mmHg)	≥110 (120)	10 (8.3%)	0.0349	36 (30%)		0.0003
	<110 (282)	2 (0.7%)		24 (8.5%)		
Proteinuria	Trace /+1(111)	0	0.0013	9 (8.1%)		<0.0001 [0.0011 (between trace/1+ and 2+/3+ proteinuria)]
	+2 (114)	1 (0.8%)		21 (18.4%)	51(25.8%)	
	+3 (84)	11 (13%)		30 (35.7%)		
Parity	Primigravida (225)	6 (2.6.%)	1.0	27 (12%)		0.2408
	Multipara (177)	6 (3.3.%)		33 (18.6%)		
Booking status	Booked (237)	1 (0.4%)	0.0649	22 (9.2%)		0.0113
	Unbooked (165)	11 (6.6%)		38 (23%)		
Socioeconomic status	Low (180)	7 (3.8%)	0.7378	32 (17.7%)		0.441
	Middle (172)	4 (2.3%)		22 (12.7%)		
	High (50)	1 (2%)		6 (12%)		
Age	<25 (172)	4 (2.3%)	0.296	39 (22.6%)		0.0001
	25-35 (160)	4 (2.5%)		7 (4.3%)		
	>35 (70)	4 (5.7%)		14(20%)		
Mode of delivery	Vaginal (212))	8(3.7%)	0.6827	39(18.3%)		0.2278
	Caesarean (190)	4 (2.1%)		21 (11%)		
Gestational age	Preterm (162)	6 (3.7%)	0.6827	36 (22.2%)		0.0327
	Term (240)	6 (2.5%)		24 (10%)		

**Table 3:** Perinatal outcome RDS: respiratory distress syndrome; FGR: fetal growth restriction; MAS: meconium aspiration syndrome; IUFD: intrauterine fetal demise

Perinatal outcome	Gestational hypertension N (%)	Mild preeclampsia N (%)	Severe preeclampsia N (%)	Eclampsia N (%)	Total N (%)
Healthy	75 (67.6 %)	60 (54.1%)	12 (8.9%)	9 (20%)	156 (38.8%)
Preterm births with RDS	18 (16.2%)	30 (27%)	45 (33.3%)	9 (20%)	102 (25.3%)
Preterm births with FGR	3 (2.7%)	9 (8.1%)	18 (13.3%)	3 (6.7%)	33 (8.2%)
Preterm births with hypoxic	9 (8.1%)	6 (5.4%)	12 (8.9%)	-	27 (6.7%)
Term births with FGR	3 (2.7%)	-	-	-	3 (0.74%)
Term births with FGR and hypoxia	3 (2.7%)	3 (2.7%)	9 (6.7%)	-	15 (3.7%)
Term births with FGR and MAS	-	-	6 (4.4%)	-	6 (1.5%)
IUFD	-	-	18 (13.3%)	15 (33.3 % )	33 (8.2%)
Neonatal death		3 (2.7%)	15 (11.1%)	9 (20%)	27 (6.7%)

## Discussion

In our study, the incidence of HDP was 7.3%. Mehta et al. found an incidence of HDP of 6.9% in the Indian population [7]. Twenty-seven point six percent (27.6%) cases in our study had gestational hypertension, another 27.6% cases had mild preeclampsia, 33.6% cases had severe preeclampsia, and 11.2% cases had eclampsia. Similar to our study, the study by Subki AH et al. reported the most prevalent hypertensive disorder as preeclampsia (54.9%) followed by gestational hypertension (29.5%) and then eclampsia (8.0%) [8]. Regarding maternal complications, in our study, 1.9% cases had HELLP syndrome, 3.7% cases had pulmonary edema, 3.9% cases had a cerebral hemorrhage, 0.7% cases had ARF, 7.4% cases had abruption placenta, and 5.7% cases had a postpartum hemorrhage. Most of the complications were seen in cases of severe preeclampsia and eclampsia. In the study by Seyom et al., 12.4% of cases had HELLP syndrome, 7% had ARF, 2% had abruption placentae, and 7% had PPH [9]. We had 12 (2.9%) maternal deaths. Bridwell et al., however, found 0.3% maternal mortality in women with HDP in their study [10]. In the present study, the cause of maternal mortality was pulmonary edema in four (33.3%) cases and cerebral hemorrhage in eight (66.6%) cases. In a study by Moodley, 50% of the maternal deaths were due to cerebral hemorrhage and 17.2% were due to pulmonary edema [11]. In our study, 16 (3.9%) women had a cerebral hemorrhage, which is much higher than that reported in a study by Miller et al., where only 0.2% of women with preeclampsia developed pregnancy-associated stroke, and in another study by Wu and colleagues, where 0.08% of HDP deliveries had a stroke [12-13]. All the women who had a cerebral hemorrhage in our study presented late to the hospital and the diagnosis of cerebral hemorrhage was made on admission and all had blood pressure (BP) > 160/110 mmHg. This reflects the poor healthcare-seeking behavior of the population. Also, since our institute is the only tertiary care center, it receives all the complicated cases of the state, which could be an additional reason for the higher incidence of cerebral hemorrhages in our study.

In our study, maternal mortality was significantly higher in women with eclampsia than with preeclampsia and in women with proteinuria of 3+. Maternal deaths were also higher, though not statistically significant, in unbooked cases and when SBP was ≥160 mmHg and DBP ≥110 mmHg. All women who had maternal deaths were treated with antihypertensive. In the Control of Hypertension In Pregnancy Study (CHIPS) trial, severe hypertension (≥160/110 mm Hg), which is considered a stroke risk threshold, developed in 27.5% of women in the tight-control group and 40.6% of the women in the less-tight control group, and the difference was highly significant, both statistically as well as clinically [14]. The UK ‘Confidential Enquiries into Maternal Deaths’ has identified that the most serious fault in the care of women who died due to stroke was the failure to recognize the severity of and to treat the severe hypertension (particularly systolic) of preeclampsia [2,15]. In preeclampsia, the severity of proteinuria has been considered by some as a predictor for poor maternal outcome [16] but others refuted the association [17]. Unbooked patients (25.9%) had more chances of developing HDP (25.9%) as compared to booked ones (6.66%) [18]. In our study, maternal mortality was more in women belonging to lower and middle-class economic status and in women more than 35 years of age. There was no association between maternal death and the mode of delivery. In our study, six maternal deaths were at a gestational age of less than 37 weeks and six deaths were at gestational age beyond that. Preeclampsia complicating pregnancy at or prior to 28 weeks are more likely to end in maternal mortality [19].

The prevalence of perinatal mortality in women with HDP has been found to be 25% in Ethiopia [20]. A study from Pakistan reported perinatal mortality of 17.5% [21]. A Norwegian study reported that 9.2% of all perinatal mortality was due to HDP [22]. In our study, the prevalence of perinatal mortality in HDP was 14.9%. Perinatal mortality was significantly more when SBP ≥160 mmHg and DBP ≥110 mmHg (p-value 0.0011, 0.0003 respectively). The study by Endeshaw G et al. also found a statistically significant association of perinatal mortality with the highest systolic BP ≥160 mmHg and diastolic BP ≥110 mmHg [23]. Perinatal deaths were more in multigravida as compared to primigravida in our study (18.6% vs 12%). Also, women with proteinuria of +2 and more had significantly higher perinatal deaths than with trace and +1 proteinuria (p-value 0.0011). However, in a study by Thangaratinam et al., proteinuria was considered a poor predictor of adverse fetal outcomes [17]. In our study, unbooked cases had higher perinatal mortality than booked cases though not statistically significantly (p-value 0.0113). In a Nigerian study too, the perinatal outcome was reported to be poorer in unbooked mothers as compared to booked mothers [24]. In our study, perinatal mortality was higher in young as well as elderly mothers. Socioeconomic condition and mode of delivery had no association with perinatal mortality. Women with preterm HDP had more perinatal mortality than term HDP though not statistically significantly (22.2% versus 10%, p-value 0.0327). A study reported that nearly three-fourths of all perinatal deaths (71%) in women with HDP occurred in those who delivered either preterm or very preterm [23]. In our study, neonatal morbidity was also more in women with severe preeclampsia, and most of the complications were related to preterm deliveries. The study by Isabela et al. also reported that neonates suffered mainly from preterm-related complications mostly in mothers with severe preeclampsia [25].

International Federation of Gynecology and Obstetrics (FIGO) screening criteria help diagnose women at risk of HDP using uterine artery Doppler and serum biomarkers in whom appropriate intervention can prevent the development of early-onset HDP [26]. In our study, the overall poor maternal and fetal outcome in HDP is more in women with severe preeclampsia and eclampsia.

## Conclusions

Pregnancies complicated by hypertensive disorders contribute substantially to the burden of maternal and perinatal morbidity and mortality, which is more in women with eclampsia and severe preeclampsia as compared to gestational hypertension and mild preeclampsia. Both maternal and perinatal mortality was more in unbooked cases. A high incidence of cerebral hemorrhage with all presenting late reflects the poor healthcare-seeking behavior of the population. Identification of the risk factors for HDP would be useful for early diagnosis in the particular patient group that requires monitoring and appropriate intervention. Besides routine antenatal screening for HDP by blood pressure measurement, the assessment of urinary proteinuria should be conducted at every antenatal visit where resources are available.
